# Feasibility of EEG-based machine learning for the objective assessment of non-Strabismic binocular vision dysfunction

**DOI:** 10.3389/fnhum.2026.1780742

**Published:** 2026-03-18

**Authors:** Zhili Lu, Xin Zuo, Qixuan Zhang, Yue Liu, Xiang Ma, Chi Zhang

**Affiliations:** 1 Department of Ophthalmology, The First Affiliated Hospital of Dalian Medical University, Dalian, Liaoning, China; 2 School of Biomedical Engineering, Faculty of Medicine, Dalian University of Technology, Dalian, Liaoning, China; 3 Faculty of Information Technology, University of Jyvaskyla, Jyvaskyla, Finland

**Keywords:** EEG, machine learning, non-strabismic binocular vision dysfunction, objective screening, vergence

## Abstract

With the increasing prevalence of prolonged near work, non-strabismic binocular vision dysfunction (NSBVD) has become a growing concern. Current diagnostic methods primarily rely on subjective symptoms and time-consuming examinations, highlighting the need for objective physiological markers. This pilot study explores the feasibility of utilizing electroencephalography (EEG) combined with machine learning as an objective, auxiliary approach for NSBVD assessment. We analyzed EEG activity in 15 NSBVD patients and 15 healthy controls during a natural viewing vergence task. Time frequency and topographic analyses were used to identify neural features associated with vergence insufficiency. The groups exhibited distinct neural patterns. Healthy controls showed strong activation in visual areas, whereas NSBVD patients displayed reduced activity, coupled with compensatory increases in frontal activity, particularly in the theta and alpha bands. A linear support vector machine (SVM) trained on these features achieved 76.67% accuracy (AUC = 0.87). These findings suggest that specific neural patterns may serve as potential biomarkers for binocular dysfunction. This study demonstrated the feasibility of objective screening, though validation in larger cohorts is needed for clinical use.

## 1 Introduction

Nowadays, rapid advances in information technology have brought about significant changes in how people work and live. With the popularity of various electronic devices such as computers and mobile phones, people tend to spend considerable time reading, surfing the web, or performing other intensive close-up visual work, which can significantly increase their visual load. Consequently, the incidence of binocular vision dysfunction (BVD) increases year by year, and more patients are suffering from visually related diseases (Jeffrey et al., 2010).

Binocular vision dysfunction can be divided into two types: strabismus and non-strabismus. Relevant epidemiological research shows that non-strabismus binocular vision dysfunction (NSBVD) has a high incidence in the general population, and about 18% of people have varying degrees of abnormal accommodation and vergence (Ai and Liu, 2015). The percentage is even higher among students due to prolonged studying and using electronic devices, requiring near vision (Dahal and Khatri, 2019; Jang and Park, 2015). This condition primarily affects visual performance, especially in close vision-related tasks, due to decreased optical system efficiency. People with NSBVD may suffer from associated symptoms such as headache, blurred vision, focusing difficulty, and so on (Dahal et al., 2021).

As for the diagnosis of NSBVD, it is still based on the nonspecific symptoms mentioned above and visual function examination results, which are susceptible to the subjective description of patients and the clinical experience of physicians (García-Muñoz et al., 2016; Shin et al., 2009). Furthermore, confirming the diagnosis and making treatment decisions are usually time-consuming, as clinical examinations require substantial time. Thus, effective and rapid diagnostic methods are urgently needed in the clinic.

While the behavioral manifestations of NSBVD are well documented, the underlying neural mechanisms remain less understood than those of other ocular conditions. Vision function is controlled by a complex cortical network that receives, integrates, and processes visual information from the retina. Extensive research using functional magnetic resonance imaging has delineated the cortical networks governing vergence eye movements. Studies by Alvarez and colleagues have consistently demonstrated that vergence control involves a widely distributed neural circuit including the frontal eye fields, parietal eye fields, and the cerebellar vermis (Alvarez et al., 2010; Jaswal et al., 2014). These regions are responsible for initiating vergence and maintaining binocular fusion.

However, the existing literature on the neurophysiology of ophthalmic diseases has largely focused on strabismus and amblyopia. For instance, Hou et al. (2016) measured the differences in neural activity in the visual cortex between strabismic amblyopes and healthy people using EEG and functional magnetic resonance imaging (fMRI). Their results showed that neural activity in the corresponding brain regions of people with amblyopia was lower than that in healthy people due to suppression or cortical reorganization. Similarly, investigations into astigmatism have analyzed how retinal input distortions create neural bias (Son et al., 2021). But the neural landscape of NSBVD differs fundamentally from these conditions. Unlike strabismus, in which fusion is often lost or suppressed to avoid diplopia, patients with NSBVD constantly struggle to maintain binocular alignment due to a fragile vergence system (Barnhardt et al., 2012; Cacho-Martínez et al., 2013). This suggests that their neural signature might be characterized by inefficient cortical recruitment or signs of neural fatigue rather than simple suppression or signal loss. Currently, the specific electrophysiological biomarkers distinguishing the effortful and unstable fusion in NSBVD patients from the comfortable fusion of healthy controls remain unknown. There is a paucity of studies using electroencephalography (EEG) to capture the high temporal dynamics of this struggle in non-strabismic patients.

In this pilot study, we propose an exploratory method that combines EEG analysis with machine learning to investigate this problem. We used a natural viewing task with red and green variable vectograms to trigger vergence eye movements. Our study compares brain topography and time-frequency results between groups. We also use a machine learning classifier to test if these brain signals could aid in diagnosis. By using a targeted feature selection process based on cluster-based permutation tests, we aim to find neural patterns with a clear biological meaning.

Since NSBVD involves difficulty maintaining eye alignment, we hypothesize that the patient group might show different brain activity in the frontal and parietal regions compared to healthy controls, especially when they try to maintain fusion. We also expect that specific power changes in the theta or alpha bands, which relate to mental effort, could serve as key features to separate the two groups. Finally, we suggest that a machine learning model based on these markers could help identify NSBVD patients, with promising future clinical use.

## 2 Materials and methods

### 2.1 Subjects

A total of 30 participants were recruited for this study, which was conducted at the First Affiliated Hospital of Dalian Medical University between September 2020 and December 2022. The cohort was divided into two groups: the NSBVD group and a healthy control group. The NSBVD group consisted of 15 patients (7 males, 8 females) aged 23 to 39 years (mean age: 27.33 ± 3.89 years). The control group comprised 15 age- and sex-matched volunteers (7 males, 8 females) aged 23 to 38 years (mean age: 26.47 ± 4.05 years).

The study protocol adhered to the tenets of the Declaration of Helsinki and was approved by the Ethics Committee of the First Affiliated Hospital of Dalian Medical University (Approval No.: PJ-KY-2019-80). Written informed consent was obtained from all participants after explaining the nature and possible consequences of the study. Participants were informed of their right to withdraw from the study at any time without penalty.

Participants in the NSBVD group were recruited from the ophthalmology clinic. The inclusion criteria are as follows: (1) Age between 18 and 45 years; (2) Absence of organic eye diseases upon examination; (3) Presence of asthenopic symptoms, including headache, blurred vision, diplopia, or difficulty maintaining focus; and (4) A confirmed diagnosis of NSBVD by a qualified optometrist based on standard clinical tests. The specific diagnostic criteria utilized for classification (e.g., Convergence Insufficiency, Convergence Excess) are detailed in Table 1.

**Table 1:** The diagnostic criteria of NSBVD.

Diagnostic critetia	Convergence insufficiency (CI)	Convergence excess (CE)	Divergence insufficiency (DI)	Divergence excess (DE)	Simple exophoria	Simple esophoria
Main symptoms occur	At near	At near	At distance	At distance		
Distance phoria			Distant esophoria 2–8∆	Distance exophoria>6 and greater than near exophoria	Distance exophoria>4∆	Distance esophoria>2
Near phoria	Near exophoria >6∆ and 4∆ greater than distance exophoria	Near esophoria >2∆			Near exophoria>7∆	Near esophoria>2∆
AC/A(∆/D)		>5:1			3–5:1	3–5:1
Near point of convergence (NPC)	>6 cm					
Positive fusional vergence (PFV)	Near PFV < 15 or<2 × near phoria			Distance PFV<10	Distance PFV<10 and near PFV<15	
Negative fusional vergence (NFV)		Near NFV<12	Distance NFV<4			Distance NFV<4 and near NFV<12

The healthy control group consisted of volunteers who met the same general criteria (age and ocular health) but reported no visual symptoms and demonstrated normal binocular vision parameters upon examination by the same optometrist.

To ensure data quality and safety, subjects were excluded if they presented with: (1) Ocular abnormalities such as strabismus or amblyopia; (2) A history of ocular or cerebral surgery; (3) Systemic or neurological disorders that could interfere with visual function or EEG recording; (4) Abnormal baseline EEG patterns; or (5) An inability to cooperate with the experimental procedures.

Before data collection, all participants were verified for eligibility. To minimize physiological artifacts, participants were instructed to ensure adequate sleep and to abstain from alcohol, caffeine (coffee or tea), and medications affecting the central nervous system for at least 24 h before the experiment. Participants were also required to keep their scalp clean to facilitate optimal EEG electrode impedance.

The exclusion criteria for all subjects are as follows: ocular abnormalities or systemic diseases such as amblyopia, strabismus, abnormal nervous system, and abnormal EEG that may affect the results of vision function examination; history of eye or brain surgery; subjects with other diseases that cannot cooperate with vision function examination; and subjects with abnormal EEGs. All subjects were determined based on the mentioned inclusion and exclusion criteria. What is more, the subjects were required to sleep well and were banned from consuming medicine, alcohol, coffee, and tea the day before the experiment. They also need to keep the scalp clean in the experiment.

### 2.2 Stimuli and procedure

Subjects were required to wear red-green glasses (red lens over the right eye and green lens over the left eye) during this experiment. The red–green variable vectograms were adopted as visual stimuli based on red or green targets that test vergence skills, utilizing similar but slightly different figures for each eye (Pena-Verdeal et al., 2019). The convergence process was achieved by moving the red sheet to the right, and the divergence process by moving the green sheet to the right. A clear vision should be guaranteed throughout the process.

**Figure 1:**
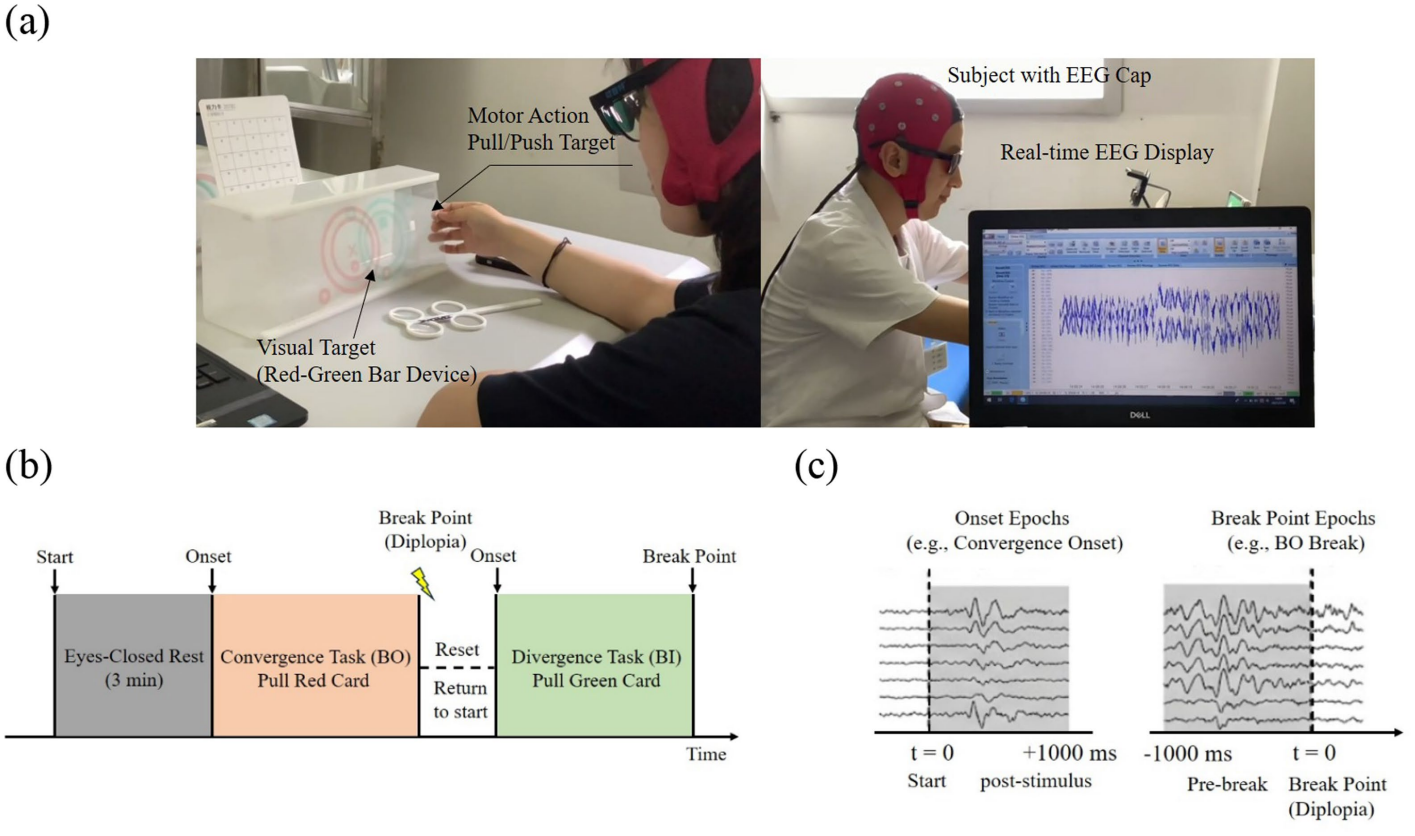
Experimental paradigm and EEG data epoching: **(a)** Experimental scene, **(b)** Experimental timeline, **(c)** Data segmentation.

**Figure 2:**
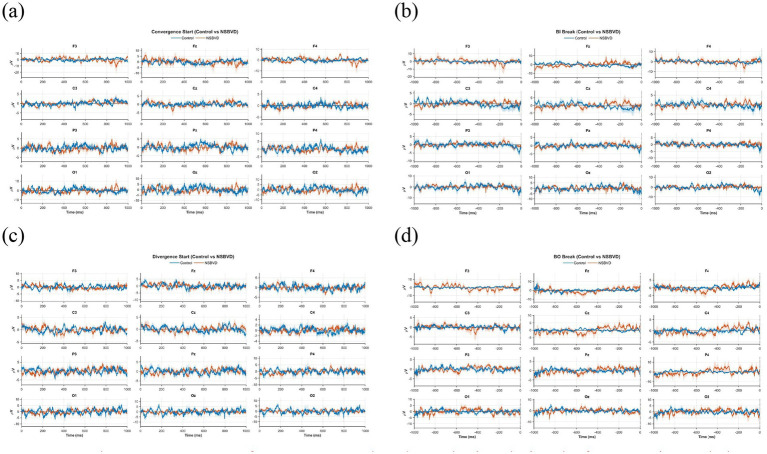
Grand average EEG waveforms across twelve electrode sites during the four experimental phases. The blue solid lines represent the Healthy Control group, and the red solid lines represent the NSBVD group. Shaded regions indicate the standard error of the mean (SEM). The x-axis represents time (ms), and the y-axis represents voltage ( $\mu_{V}$ ). **(a)** Convergence Start (0 to 1,000 ms): At the onset of convergence, the NSBVD group appears to exhibit unstable, high-amplitude fluctuations, particularly in the frontal regions (F3, F4). This pattern differs from the relatively stable and controlled activation observed in the control group. **(b)** BO Break (−1,000 to 0 ms): As the eyes approach the breaking point of fusion (Base-Out Break), the NSBVD group displays a noticeable increase in wave amplitude across the Frontal (F3, Fz, F4) and Occipital (O1, Oz, and O2) channels. **(c)** Divergence Start (0 to 1,000 ms): Unlike convergence, the initiation of divergence in NSBVD patients is characterized by reduced signal amplitudes and a lack of distinct rhythmic patterns. **(d)** BI Break (−1,000 to 0 ms): Before the divergence break (Base-In Break), patients show relatively weak and non-specific neural activity compared to the controls.

Figure 1a shows the experiment scene. The experiment was conducted in a quiet, well-lit room. Subjects requiring vision correction were instructed to wear their full correction glasses underneath the red-green goggles. The vectogram device was positioned on an inclined stand at a viewing distance of 40 cm from the subject. The green sheet was on top of the red sheet. A 32-channel Brain Products electrode cap, arranged according to the international 10–20 system (see Figure 2), was used to collect EEG signals, and ANT Neuro devices were used for data acquisition. The sampling rate was set to 500 Hz. The impedance between each electrode and the scalp was maintained below 30 kΩ.

Before the experiment, the procedure was explained in detail to the subjects, and their understanding was confirmed. A 1-min practice session was conducted to familiarize participants with the task, after which the formal experiment officially started. The experimental timeline is outlined in Figure 1b. Subjects began by closing their eyes and resting for 3 min. The convergence block then commenced. Subjects opened their eyes and pulled the red sheet to the right at a uniform speed of approximately two prism diopters per second (2∆/s). During this process, subjects were instructed to maintain binocular fusion of the target. They were required to stop moving the sheet and report to the experimenter as soon as fusion was lost and diplopia occurred. The value recorded at this moment was defined as the convergence breakpoint (BO Break). After the red sheet was returned to its initial position, the divergence block was performed. The green sheet was moved to the right at a speed of about 2∆/s until diplopia was perceived. Subjects stopped the movement and reported immediately, allowing the divergence breakpoint (BI Break) to be recorded. EEG signals were continuously acquired throughout the experiment and subsequently segmented into epochs as depicted in Figure 1c.

### 2.3 Analysis

The EEG analysis process consists of four parts: preprocessing, brain topography calculation, wavelet-based time-frequency analysis, and statistical analysis for the power of different rhythms. This subsection describes how we dealt with the data.

#### 2.3.1 Preprocessing

EEG data preprocessing was performed using the EEGLAB toolbox in MATLAB. First, the raw data were re-referenced to the global average reference. To eliminate power line interference, a 50 Hz bandstop filter was applied. Subsequently, a bandpass filter with a 0.5–31.25 Hz was used to retain the frequency bands of interest. Bad channels were identified and corrected using spherical spline interpolation. Finally, to remove ocular and muscular artifacts, we used independent component analysis (ICA) based on the RunICA algorithm. Artifactual components (e.g., eye blinks, muscle noise) were identified and rejected using a hybrid approach of automatic classification (ICLabel) and manual visual inspection of component topographies and time courses (Stewart et al., 2014).

#### 2.3.2 EEG waveform analysis

To characterize the cortical dynamics associated with natural vergence eye movements, we computed grand average EEG waveforms for both the Control and NSBVD groups. Unlike traditional paradigms using discrete sensory stimuli, this study employed a natural viewing task. Therefore, the analysis focused on the continuous evolution of cortical potentials time-locked to specific behavioral events: the initiation of vergence (Start) and the loss of fusion (Break).

The analysis targeted four distinct experimental phases: Convergence Start, Convergence Break, Divergence Start, and Divergence Break. For each phase, the EEG data were segmented and averaged across subjects to generate group-level waveforms. The temporal windows for visualization were defined to align with the dynamics of the vergence task. For the “Start” conditions, waveforms were analyzed from 0 ms to 1,000 ms relative to movement onset, capturing the neural processes underlying vergence initiation. Conversely, for the “Break” conditions, the analysis window was set from 1,000 ms preceding the break point up to 0 ms (the moment of fusion loss). This window was selected to examine sustained cortical effort and the subsequent failure of the fusion maintenance mechanism.

To visually represent the inter-subject variability and the reliability of the mean estimates, shaded error bands corresponding to the standard error of the mean (SEM) were plotted around each waveform.

#### 2.3.3 Brain topography analysis

Following the preprocessing, the artifact-free EEG data were subjected to topographical analysis to map the spatial distribution of cortical activity associated with vergence dynamics. The segmentation of EEG epochs was rigorously time-locked to specific behavioral events across the four experimental conditions: Convergence Start, Convergence Break, Divergence Start, and Divergence Break.

For the “Start” conditions (Convergence and Divergence), EEG epochs were extracted from a temporal window spanning 0 ms to 1,000 ms relative to the initiation of the vergence movement. This window was selected to capture the neural processes underlying the recruitment of oculomotor resources. Conversely, for the “Break” conditions, epochs were extracted from the 1,000 ms interval immediately preceding the fusion break point (i.e., −1,000 ms to 0 ms). This backward extraction strategy enabled examination of the sustained cortical effort leading up to the loss of binocular fusion.

To generate the brain topographies, the segmented trials were first averaged across all trials for each subject to obtain individual subject means. Subsequently, a grand average was computed across all subjects within the Control and NSBVD groups, respectively. These group-level potential values were then mapped onto a standard 2D scalp surface using spherical spline interpolation.

#### 2.3.4 Time-frequency analysis

Following the topographical examination, we employed a time-frequency analysis to capture the dynamic oscillatory changes associated with vergence eye movements. Unlike the static spectral analysis provided by the Fourier transform, the wavelet transform offers an optimal balance of resolution for non-stationary signals like EEG, preserving both temporal and spectral information.

Consistent with the previous sections, the EEG data were segmented into distinct phases of the vergence task. For the Convergence Start and Divergence Start conditions, the analysis focused on the post-movement epoch ranging from 0 ms to 1,000 ms relative to the stimulus onset. For the Convergence Break and Divergence Break conditions, the analysis targeted the preparatory and failure phases, defined as the interval from 1,000 ms before the break point up to the break onset (0 ms). To minimize edge effects inherent in the convolution process, the actual computation window was extended bilaterally during the transformation and subsequently trimmed to the regions of interest.

We utilized the Continuous Wavelet Transform (CWT) to decompose the EEG signal into time-frequency representations. The Complex Morlet wavelet was selected as the mother wavelet due to its biological plausibility and superior capacity to separate phase and amplitude information. In this study, we got the wavelet time-frequency diagram by following the steps listed below (Jia et al., 2022).

1) Suppose *a* is the scaling factor, *b* represents the time shift factor, ψ(t) is the mother wavelet, and the continuous wavelet transform of the EEG signal *s*(*t*) is defined as

$$W_{T}=\langle s,\psi_{a,b}\rangle ={|a|}^{-\frac{1}{2}}\int_{R}s(t)\;\bar{\psi (\frac{t-b}{a})}\mathrm{dt}$$ (1)

Where $\bar{\psi (t)}$ is the complex conjugate of ψ(t) , $\langle s,\psi_{a,b}\rangle$ is the inner product of *s* and ψ .

2) The sampling rate of EEG is $f_{s}$ , $F_{c}$ is the central frequency of ψ(t) , then the frequency $F_{a}$ corresponding to *a* is

$$F_{a}=F_{c}\times f_{s}/a$$ (2)

3) According to Equation 2, scale series should be defined as follows to obtain $F_{a}$ , which is an arithmetic sequence

c/totalscal,c/(totalscal−1),⋯,c/2,c (3)

Where *totalscal* is the length of the scale series used in the wavelet transformation of the signal; *c* is a constant.

4) Based on the sampling theorem and Equation 2, scale c/totalscal corresponds to frequency $f_{s}/2$ , then

$$c=2\times F_{c}\times \text{totalscal}$$ (4)

Substitute Equation 4 into Equation 3 to determine the needed scale series *a*.

5) In the last step, Equation 2 is applied to calculate the wavelet coefficients $W_{T}$ with (ψt) and *a* in Equation 1. Then, transform the scale series into a frequency series $F_{a}$ , thus, we finally obtain the wavelet time-frequency diagrams.

#### 2.3.5 Statistical analysis

Statistical analysis was performed using SPSS 25.0 software to compare baseline data and EEG power differences between the NSBVD and the healthy groups. Analysis of EEG power differences was performed between the NSBVD and the healthy control groups using MATLAB R2021a.

For the baseline data, we first checked if the data distribution was normal using the Shapiro–Wilk test. For normally distributed data, we used independent samples t-tests. For data that were not normally distributed, we used the Mann–Whitney U test. For categorical data, such as sex ratio, we used the chi-square test. All clinical data are shown as Mean ± Standard Deviation (SD). A *p*-value less than 0.05 was considered significant.

To evaluate the differences in EEG signals, we used a non-parametric cluster-based permutation test. This method is highly effective for addressing the multiple-comparison problem in high-dimensional EEG data. We focused on the theta (4–8 Hz), alpha (8–13 Hz), and beta (13–30 Hz) bands. The analysis followed these steps: First, we calculated an independent samples t statistic for each data point to measure the difference between the two groups. Points with a *p*-value less than 0.05 were kept, and neighboring significant points were grouped into clusters. For each cluster C , we calculated a cluster mass $M_{C}$ by summing the t values of all points inside that cluster Equation 5:


$$M_{C}=\Sigma_{\left(t,f\in c\right)}\;t_{\text{value}}\;(t,f)$$ (5)

To determine the statistical significance of these clusters, we generated a null distribution using the Monte Carlo method. We randomly shuffled the group labels between the Control and NSBVD groups 5,000 times. For each iteration, we recorded the maximum cluster mass to build a reference distribution. The observed cluster mass was then compared against this distribution. A cluster was considered significant if its *p*-value was less than 0.05 based on a two-tailed test. This method strictly controls the error rate while keeping high sensitivity.

#### 2.3.6 Machine learning classification framework

To evaluate whether the identified brain signals could help in diagnosing NSBVD, we built a machine learning classification framework. We used a targeted feature engineering strategy to connect group-level statistical results with subject-level diagnosis. Instead of using all features from every channel and frequency band, which might introduce noise and lead to overfitting with small sample sizes, we only used data from the regions of interest and frequency bands that showed significant differences in the cluster-based permutation tests. This method ensures that the classification model is based on neural signals with clear biological meaning.

To comprehensively evaluate the data structure, we employed three distinct classifiers. First, we selected the support vector machine (SVM) with a linear kernel for its proven robustness in small-sample scenarios and its ability to maximize the margin between classes. Second, Linear Discriminant Analysis (LDA) was included as a standard baseline to assess linear separability, given its computational efficiency. Finally, K-Nearest Neighbors (KNN, with K = 3) was chosen for its non-parametric nature, allowing for the detection of local data structures without assuming specific underlying distributions.

The workflow followed four specific steps. First, data splitting, in each round, one subject was kept as the test set while the remaining N-1 subjects formed the training set. Second, standardization, where we calculated the Z score based only on the training set and then applied it to the test subject. This maintains independence between the sets. The formula for Z-score normalization is defined as Equation 6:


$$z=\frac{x-\mu }{\sigma }$$ (6)

Where x is the raw feature value, μ is the mean of the training set, and σ is the standard deviation of the training set. Third, feature ranking, where we ranked features within the training set using independent t-tests and selected the top discriminative features for model training. Finally, in the evaluation step, the trained model was tested on the unseen subject. This nested process ensures that feature selection and model optimization remain completely blind to the test data, providing an unbiased estimate of how well the model works on new data.

## 3 Results

### 3.1 Demographic and clinical baseline analysis

Statistical analysis was performed to evaluate the baseline comparability and clinical distinctions between the two groups. The study included 30 participants, divided equally into the NSBVD group (*n* = 15) and the healthy control group (*n* = 15).

Regarding demographic characteristics, the groups were well-matched. The mean age of the NSBVD group was 27.33 ± 3.89 years, while that of the healthy control group was 26.47 ± 4.05 years. A Mann–Whitney U test confirmed no statistically significant difference in age between the groups (Z = 1.195, *p* = 0.250). Similarly, the sex distribution was identical in both groups, with each group consisting of 7 males (46.7%) and 8 females (53.3%). A Chi-Square test yielded a *p*-value of 1.000, indicating no difference in sex composition. These results demonstrate that potential confounding factors related to age and sex were effectively controlled.

In terms of clinical binocular function, the NSBVD group exhibited specific deficits consistent with the diagnosis. Significant differences were observed in the Near Point of Convergence (NPC) and Positive Fusional Vergence (PFV). The NSBVD group showed a significantly reduced NPC (6.33 ± 2.72 cm) than the control group (4.33 ± 0.72 cm; *p* = 0.014). Furthermore, the Positive Fusional Vergence was significantly lower in the NSBVD group (18.20 ± 6.01 ∆) than in the control group (26.00 ± 3.51 ∆; *p* < 0.001). Other optometric parameters, such as distance and near phoria and the AC/A ratio, showed no statistically significant differences between the groups (*p* > 0.05). Detailed demographic and clinical data are summarized in Table 2.

**Table 2:** Baseline data and clinical characteristics of the NSBVD and healthy control groups.

Variable	NSBVD group (*n* = 15)	Control group (*n* = 15)	Test statistic	*P-*value
Sex (male/female)	7/8	7/8	χ^2^ = 0	1.000
Age (years)	27.33 ± 3.89	26.47 ± 4.05	Z = 1.195^a^	0.250
Distance phoria (∆)	−0.93 ± 2.93	−1.20 ± 0.99	t = 0.334^b^	0.742
Near phoria (∆)	−5.53 ± 6.43	−3.23 ± 1.83	t = −1.335^b^	0.200
AC/A (∆/D)	3.97 ± 1.91	4.25 ± 0.46	t = −0.563^b^	0.582
NPC (cm)	6.33 ± 2.71	4.33 ± 0.75	t = 2.756^b^	0.014*
PFV (∆)	18.20 ± 6.71	26.00 ± 3.36	t = −4.024^b^	<0.001*
NFV (∆)	18.67 ± 6.92	18.73 ± 1.83	t = −0.036^b^	0.972

Data are presented as mean±standard deviation (SD) or number. NSBVD, Non-strabismus Binocular Vision Dysfunction; ∆, Prism Diopters; D, Diopters; AC/A, Accommodative Convergence to Accommodation ratio; NPC, Near Point of Convergence; PFV, Positive Fusional Vergence; NFV, Negative Fusional Vergence. Negative values in phoria indicate exophoria, positive values indicate esophoria.

a Mann–Whitney U test (Standardized Test Statistic reported).

b Welch’s *t*-test used due to unequal variances. * Indicates statistical significance (*p* < 0.05).

### 3.2 Preprocessed EEG signals

Previous studies have indicated that the frontal, parietal, and occipital cortices are involved in vergence eye movements (Alvarez et al., 2010). In this part, we selected 12 electrodes in the relevant brain areas to observe the preprocessed EEG signals. They are F3, Fz, F4 in the frontal region, C3, Cz, C4 in the central region, P3, Pz, P4 in the parietal region, and O1, Oz, O2 in the occipital region. Visual inspection of the grand-average waveforms revealed distinct activation patterns distinguishing the NSBVD patients from healthy controls. These differences reflect alterations in the neural strategies employed during dynamic binocular coordination, as illustrated in Figure 2.

Figures 2a,c display the cortical potential shifts during the initiation phase of convergence and divergence (0–1,000 ms). In the Control group, the waveforms exhibited robust amplitude modulations, particularly in the Occipital and Frontal regions. This activity likely reflects the efficient recruitment of cortical networks necessary to process retinal disparity cues and generate the motor command for vergence initiation.

Conversely, the NSBVD group showed attenuated cortical responses during this critical initiation phase. The amplitude of the neural excursions in both posterior sensory and anterior motor regions was noticeably reduced relative to the controls. This reduction suggests a potential deficit in neural recruitment or a lower gain in the sensorimotor loop. The weaker cortical engagement in patients implies that the neural drive required to initiate precise vergence movements is suboptimal, potentially contributing to the delayed or unstable eye movements often observed in this population.

Figures 2b,d illustrate the temporal evolution of cortical activity leading up to the fusion break (1000–0 ms). This period characterizes the struggle to maintain binocular alignment under increasing disparity load.

The Control group demonstrated sustained negative or positive potential shifts (depending on the electrode site) leading up to the break point, particularly in the central and Parietal channels. This sustained activity likely reflects continuous top-down cognitive effort and oculomotor control exerted to preserve fusion against the natural limit. However, the NSBVD group exhibited a markedly different pattern. The waveforms appeared flatter and lacked the progressive build-up observed in controls. This absence of sustained neural engagement prior to the break suggests a premature failure of the fusion maintenance mechanism. It indicates that in NSBVD patients, cortical resources required to sustain vergence alignment may be exhausted earlier or insufficiently mobilized, leading to a breakdown in binocular vision.

Collectively, these findings indicate that the functional deficits in NSBVD are not limited to the peripheral oculomotor system but extend to cortical processing. The altered waveform dynamics reflect a systemic inefficiency in both the initiation of vergence movements and the sustained neural effort required for fusion maintenance.

### 3.3 Brain topography

**Figure 3:**
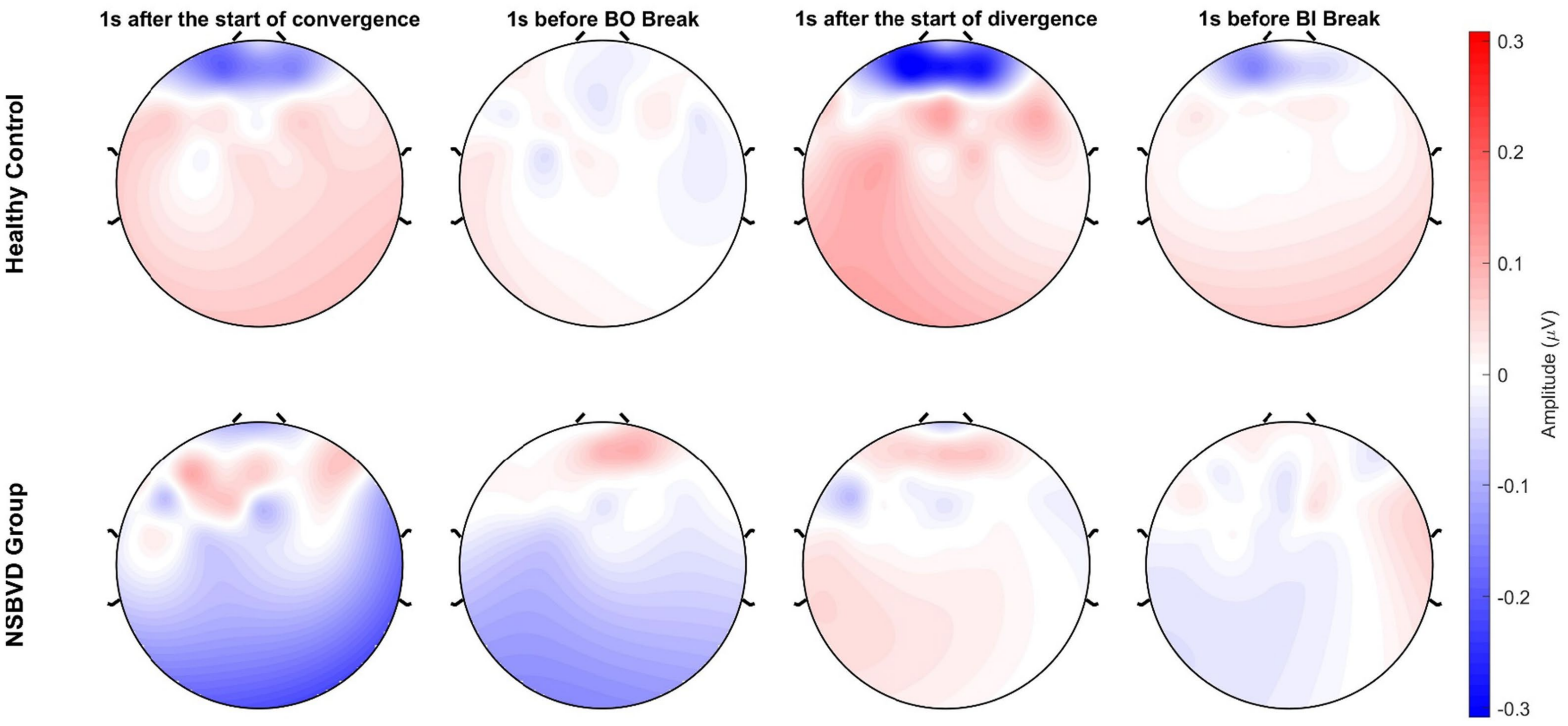
Topographical EEG maps for Healthy Control (top row) and NSBVD (bottom row) groups across four experimental stages. Red and blue indicate positive and negative potentials ( $\mu_{V}$ ), respectively. A distinct polarity reversal is observed between the groups. While controls show positive activation (red) in parietal-occipital regions, patients display negative potentials (blue) in the same areas.

Figure 3 depicts the topographical distribution of scalp electrical potentials across the four experimental conditions. Standardized color scales were applied to facilitate a rigorous comparison between the two groups.

In the convergence block, the analysis revealed a distinct dichotomy in neural polarity. The healthy control group exhibited widespread, robust positive activation within the first second of task onset, predominantly localized to the parietal and occipital regions. This activation profile indicates an efficient engagement of the dorsal visual stream, reflecting the normative physiological response required for vergence control. Conversely, the NSBVD group demonstrated a pervasive negative potential shift that emerged at onset and persisted throughout the maintenance phase, culminating in the break of fusion. This pronounced inversion from the positivity observed in controls to the sustained negativity in patients suggests a fundamental alteration in neural processing strategies. Such a phenomenon likely points to a compensatory inhibitory mechanism or a maladaptive shift in neural gain control, necessitated by the excessive cognitive effort required to accommodate high vergence demands.

Turning to the divergence block, the disparity between groups transitioned from a reversal of polarity to a marked attenuation in neural responsiveness. The NSBVD group showed a profile characterized by flattened topography and significantly dampened cortical responses throughout the task. Conversely, the healthy control group demonstrated dynamic spatiotemporal modulation that culminated in a surge of activation in the frontal and parietal areas immediately preceding the break of fusion. This amplification in the late stage likely reflects the recruitment of cognitive control networks essential for sustaining binocular alignment as disparity increases. The absence of this protective neurophysiological response in the NSBVD group suggests a potential deficit in the mobilization of cortical resources required to maintain fusion under divergent stress.

### 3.4 Wavelet time-frequency analysis

**Figure 4:**
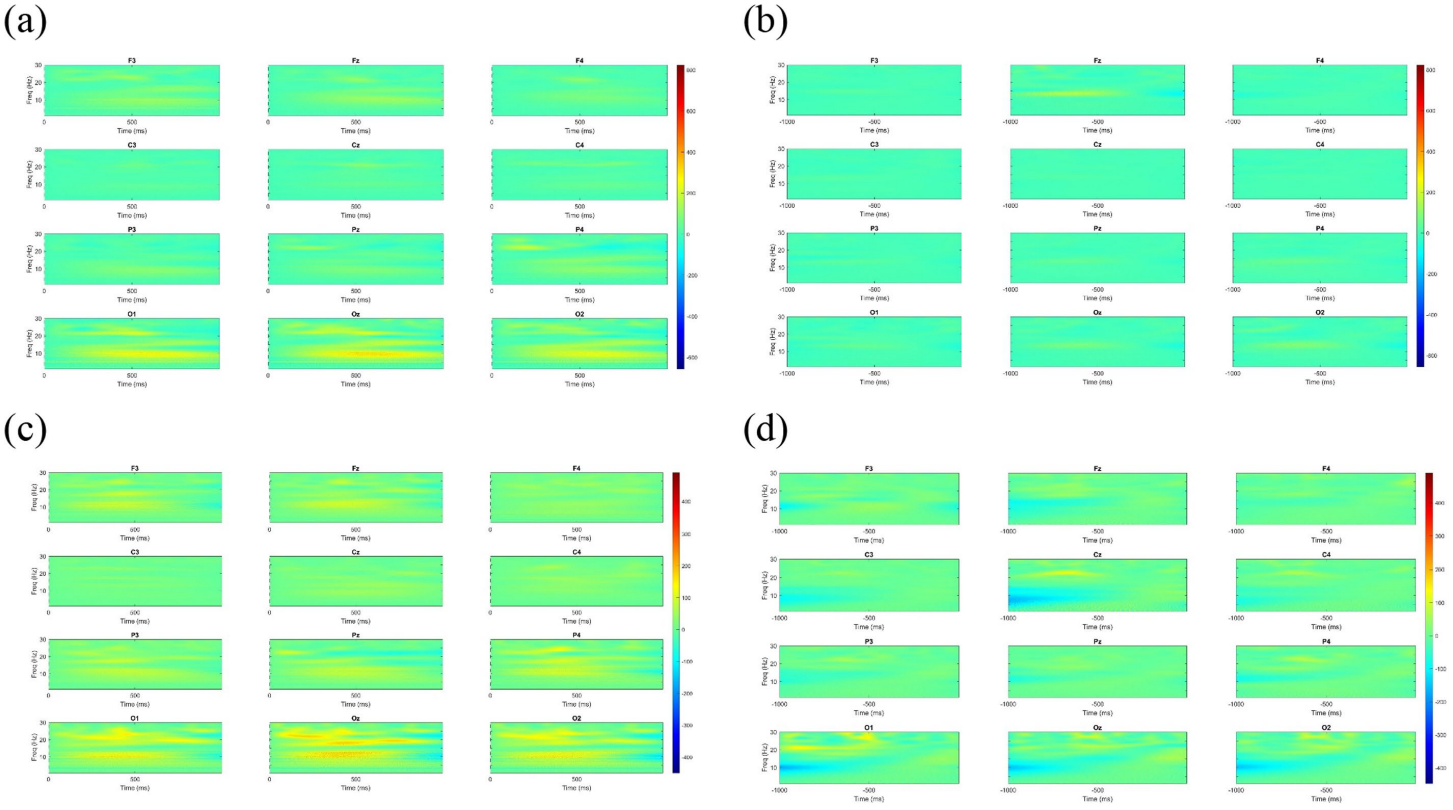
Time-frequency spectral power analysis (Spectrograms) of Healthy Controls across the four experimental phases. The x-axis represents time (ms), and the y-axis represents frequency (Hz). The color scale indicates spectral power, with warmer colors (red/yellow) denoting higher energy and cooler colors (blue) denoting lower energy. **(a)** Convergence Start and **(c)** Divergence Start: Following stimulus onset (0–1,000 ms), healthy controls exhibit robust power increases in the alpha (8–13 Hz) and beta (13–30 Hz) bands. This activation is most prominent in the frontal (Fz) and posterior (Pz, Oz) regions. **(b)** BO Break and **(d)** BI Break: Approaching the fusion break (−1,000–0 ms), sustained high-frequency activity is observed.

**Figure 5:**
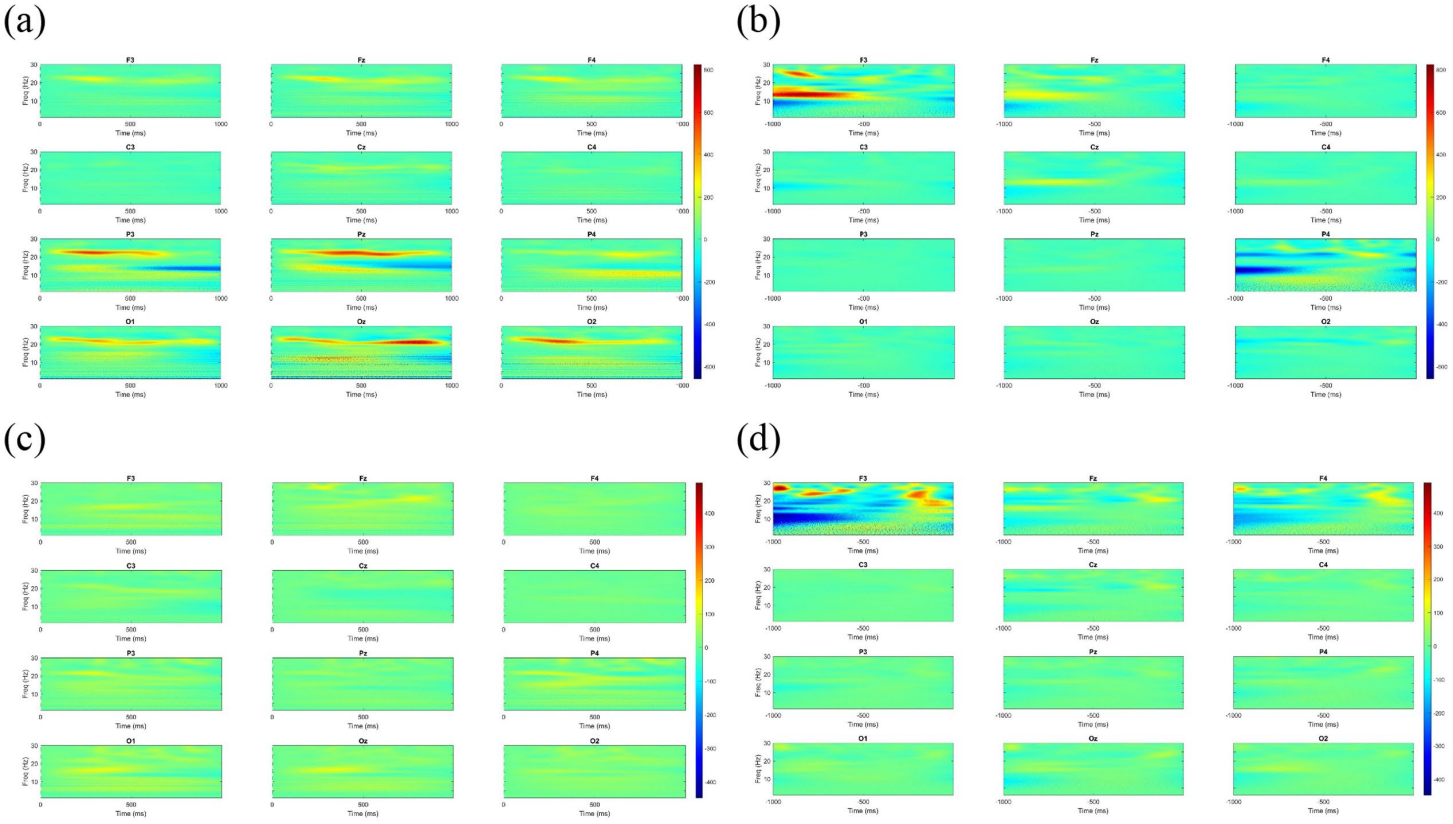
Time-frequency spectral power analysis (Spectrograms) of the NSBVD group across the four experimental phases. The axes and color scale correspond to those in Figure 4. Warmer colors indicate higher spectral power, while cooler colors indicate lower power. **(a)** Convergence Start and **(c)** Divergence Start: In contrast to the robust activation seen in controls, patients exhibit generalized hypoactivation (predominantly green/blue backgrounds), particularly in the posterior visual cortex (Pz, Oz). **(b)** BO Break and **(d)** BI Break: Approaching the fusion break, erratic high-frequency power surges are observed, specifically isolated in the left frontal region (F3).

Figures 4, 5 illustrate the wavelet time-frequency diagrams for the healthy control and NSBVD groups, respectively. These figures display the spatiotemporal distribution of spectral power across the twelve electrode sites during the convergence and divergence experimental blocks. The analysis specifically focuses on the temporal dynamics relative to the task initiation and the critical phase preceding the break of fusion.

During convergence, in the healthy control group, initiation of convergence (Figure 4a) was characterized by a well-regulated and moderate distribution of spectral power. This activation appeared widely distributed across the scalp but maintained a controlled intensity, particularly within the theta and alpha frequency ranges. As participants approached the point of fusion loss or BO Break (Figure 4b), the spectral profile remained stable, with minimal energy fluctuation. This pattern suggests a state of neural efficiency wherein the healthy visual system sustains binocular alignment with optimal cortical resource expenditure and minimal oscillatory noise.

Conversely, the NSBVD group presented a markedly different neurophysiological profile. Upon the start of convergence (Figure 5a), specific regions exhibited prominent localized bursts of elevated spectral power. Crucially, as the task progressed into the critical BO Break phase (Figure 5b), this group exhibited intense, irregular surges in low-frequency power, most notably observed in the frontal and parietal channels. This distinct elevation in spectral energy likely reflects a compensatory neural mechanism or a state of cortical instability. It implies that NSBVD patients may require excessive recruitment of neural substrates to compensate for increasing disparity load, ultimately leading to a less efficient processing strategy compared to controls.

### 3.5 Statistical analysis

Guided by the initial inspection of topographic maps and time-frequency distributions (Section 3.4), which suggested widespread cortical activation patterns during the vergence task, statistical analysis was focused on two key regions of interest (ROIs): the frontal region (F3, Fz, and F4) and the Parieto-Occipital region (P3, Pz, P4, O1, Oz, and O2). The aggregation of parietal and occipital electrodes into a single Parieto-Occipital ROI was motivated by the aim of capturing the integrated activity of the dorsal visual stream—a pathway crucial for disparity processing—while potentially mitigating volume conduction effects and enhancing the signal-to-noise ratio. Furthermore, the analysis targeted the theta (4–8 Hz), alpha (8–13 Hz), and beta (13–30 Hz) frequency bands, as preliminary observations indicated that these rhythms exhibited distinct modulations in response to disparity stress.

**Figure 6:**
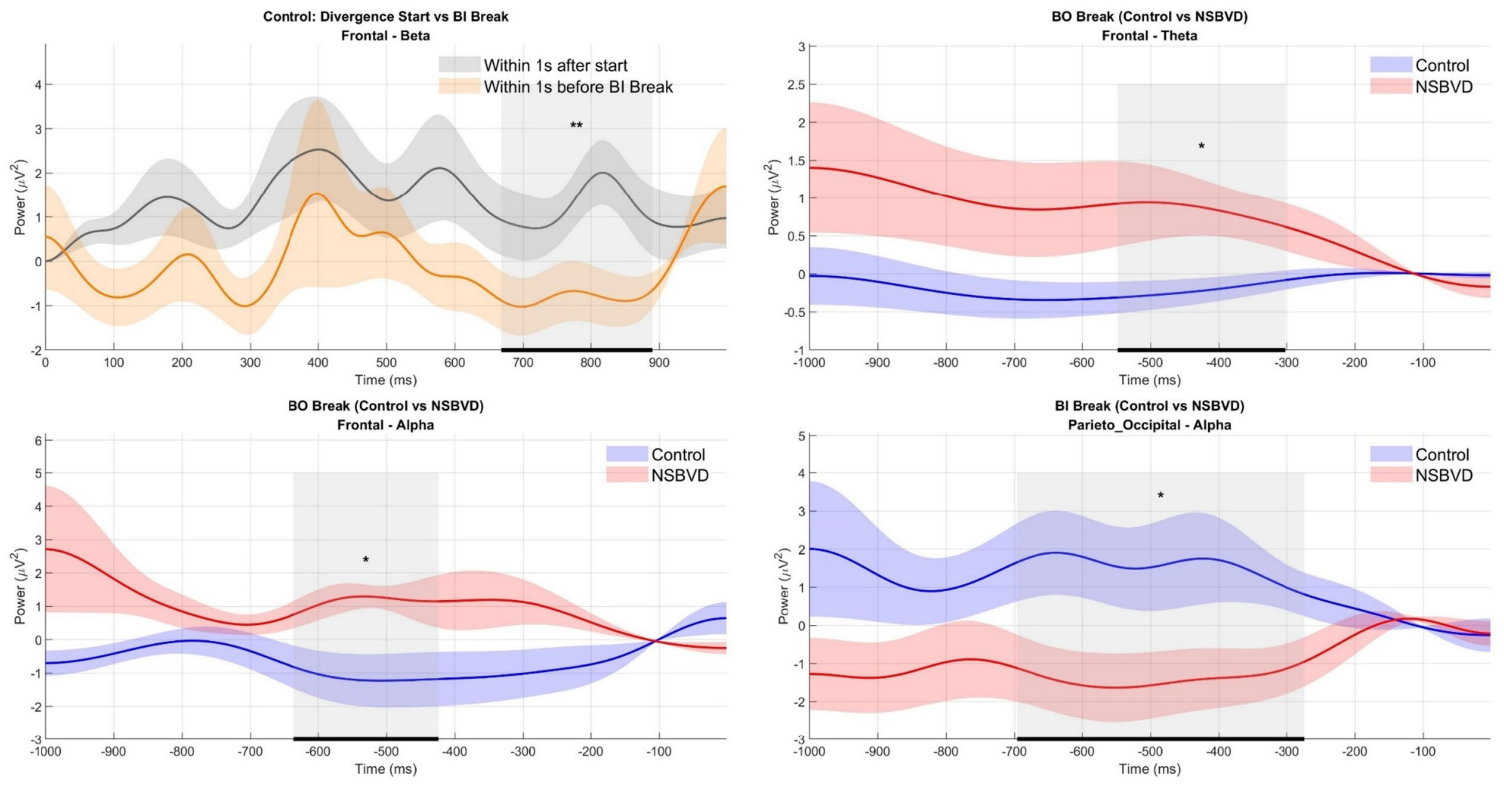
Statistical comparisons of EEG power ratios across experimental conditions and groups. Shaded vertical bars indicate time windows with statistically significant differences (*p* < 0.05). (Top-Left). In healthy controls, frontal beta power is significantly higher during divergence initiation compared to the break phase. (Top-Right and Bottom-Left). During the BO Break, the NSBVD group exhibits significantly elevated frontal theta and alpha power compared to controls. (Bottom-Right). Conversely, during the BI Break, controls maintain significantly higher Parieto-Occipital Alpha power, whereas patients show reduced activity.

The detailed statistics of the significant clusters identified by the cluster-based permutation tests are summarized in Table 3, and the between-group comparisons of EEG power ratios are shown in Figure 6.

**Table 3:** Summary of significant clusters revealed by cluster-based permutation tests.

Comparison	Type	ROI	Band	Time window (ms)	Cluster mass (*t*_sum_)	*P*-value
Control: Div. Start vs. BI Break	Within-group	Frontal	Beta	668–890	321.35	**0.01**
Between: BO Break (Ctrl vs. NSBVD)	Between-group	Frontal	Theta	−548 to −302	303.31	**< 0.05**
Between: BO Break (Ctrl vs. NSBVD)	Between-group	Frontal	Alpha	−636 to −424	273.77	**< 0.05**
Between: BI Break (Ctrl vs. NSBVD)	Between-group	Parieto-occipital	Alpha	−696 to −274	488.05	**< 0.05**

Time windows for “Between-Group” comparisons are relative to the fusion break point (0 ms), where negative values indicate the maintenance phase preceding the break. “Div.”, Divergence; “BO”, Convergence limit; “BI”, Divergence limit. Significant differences are thresholded at *p* < 0.05 (cluster-corrected). t_sum_ represents the cluster mass. Bold values indicate significant differences.

In the Control group, the analysis of the divergence task indicated a notable modulation in the frontal region. Specifically, beta band power exhibited a statistically significant cluster (Cluster Mass = 321.35, *p* = 0.010) spanning the time window from 668 ms to 890 ms after stimulus onset. This elevation in beta activity as subjects approached the fusion limit might reflect an increased demand for top-down cognitive control and attentional monitoring required to sustain fusion against increasing disparity stress. Notably, this adaptive modulation was not statistically evident in the NSBVD group, potentially signaling a deficit in dynamically mobilizing cortical resources during high-demand visual processing.

Regarding the between-group comparisons at the critical phase of fusion breakage, distinct neural strategies appeared to be engaged. During the convergence task (BO Break), significant differences emerged in the frontal cortex within the time windows preceding the break point. Statistical analysis identified significant clusters in both the theta band (*p* = 0.045, Cluster Mass = 303.31) and the Alpha band (*p* = 0.023, Cluster Mass = 273.77), occurring approximately −636 ms to −302 ms relative to the break. Since frontal theta oscillations are frequently associated with cognitive effort and error processing, while Alpha activity is often linked to inhibitory control mechanisms, these alterations may imply that NSBVD patients exhibit signs of neural inefficiency or aberrant compensatory processing in anterior cortical networks when confronting convergence failure.

Furthermore, during the divergence task (BI Break), a prominent difference was observed in the posterior brain regions. A significant cluster in the Alpha band was localized to the Parieto-Occipital region (*p* = 0.015, Cluster Mass = 488.05), extending from −696 ms to −274 ms before the break. Given the role of the parieto-occipital cortex in visual sensory integration, this variation in Alpha power may suggest that the fundamental processing of divergent disparity and the modulation of visual cortical excitability are compromised in patients with NSBVD. Collectively, these electrophysiological findings suggest that NSBVD is characterized by a dual dysfunction involving both posterior sensory processing deficits and anterior executive control abnormalities when the binocular system is driven to its physiological limits.

### 3.6 Machine learning classification performance

Based on the targeted feature engineering approach described in the Methods, we evaluated the classification performance of the SVM, LDA, and KNN models. Table 4 summarizes the comprehensive performance metrics derived from the Nested LOOCV procedure.

Among the evaluated models, the linear SVM classifier exhibited the most robust overall performance, achieving a classification accuracy of 76.67% and the highest Area Under the Curve (AUC) of 0.8711. In the context of clinical screening, sensitivity is often considered a paramount metric. The SVM model demonstrated a superior sensitivity of 80.00%, indicating a strong ability to correctly detect NSBVD cases. While the LDA model achieved the highest specificity (80.00%) and precision (76.92%), its sensitivity (66.67%) was comparatively lower than that of the SVM. Conversely, the non-parametric KNN model lagged behind, achieving 63.33% accuracy and an AUC of 0.6800.

**Figure 7:**
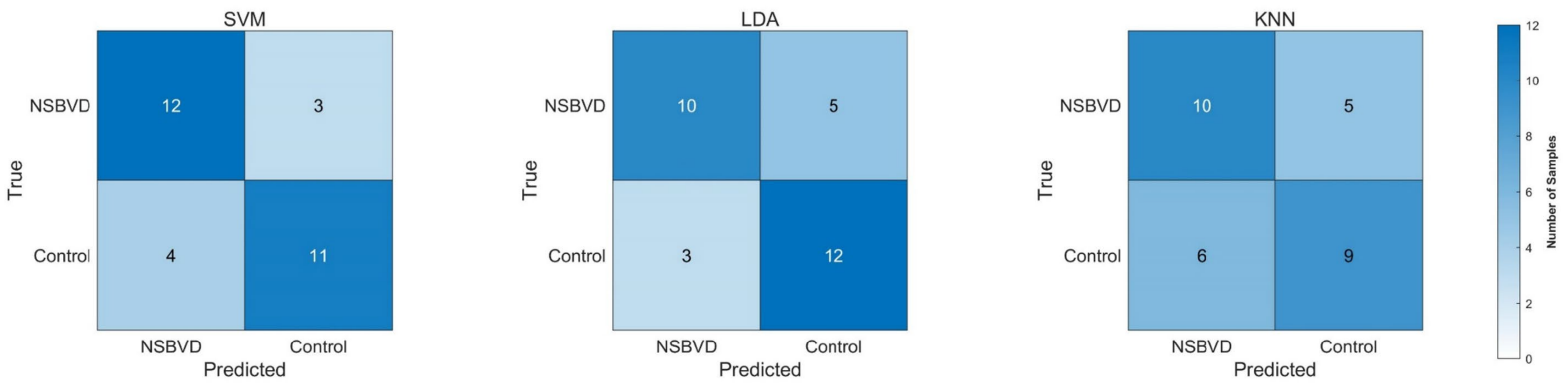
Confusion matrices comparing SVM, LDA, and KNN classification models.

Figure 7 (Confusion Matrices) provides a detailed breakdown of the classification outcomes. The confusion matrix for the SVM classifier indicates balanced classification, correctly identifying the majority of true positive cases (NSBVD patients) with relatively few false negatives. Conversely, the KNN classifier showed a higher rate of misclassification, particularly in distinguishing healthy controls, as evidenced by lower counts along the true- negative diagonal.

**Table 4:** Diagnostic performance of machine learning classifiers based on targeted physiological features.

Classifier	Accuracy	Sensitivity	Specificity	Precision	F1-Score	AUC
SVM	**76.67%**	**80.00%**	73.33%	75.00%	**0.7742**	**0.8711**
LDA	73.33%	66.67%	**80.00%**	**76.92%**	0.7143	0.7822
KNN	63.33%	66.67%	60.00%	62.50%	0.6452	0.6800

Performance metrics were obtained using a Nested Leave-One-Out Cross-Validation (Nested LOOCV) procedure to ensure unbiased estimation. SVM: Support Vector Machine (linear kernel); LDA: Linear Discriminant Analysis; KNN: K Nearest Neighbors (K = 3). AUC, Area Under the Receiver Operating Characteristic Curve. Bold values indicate the best performance in each metric.

**Figure 8:**
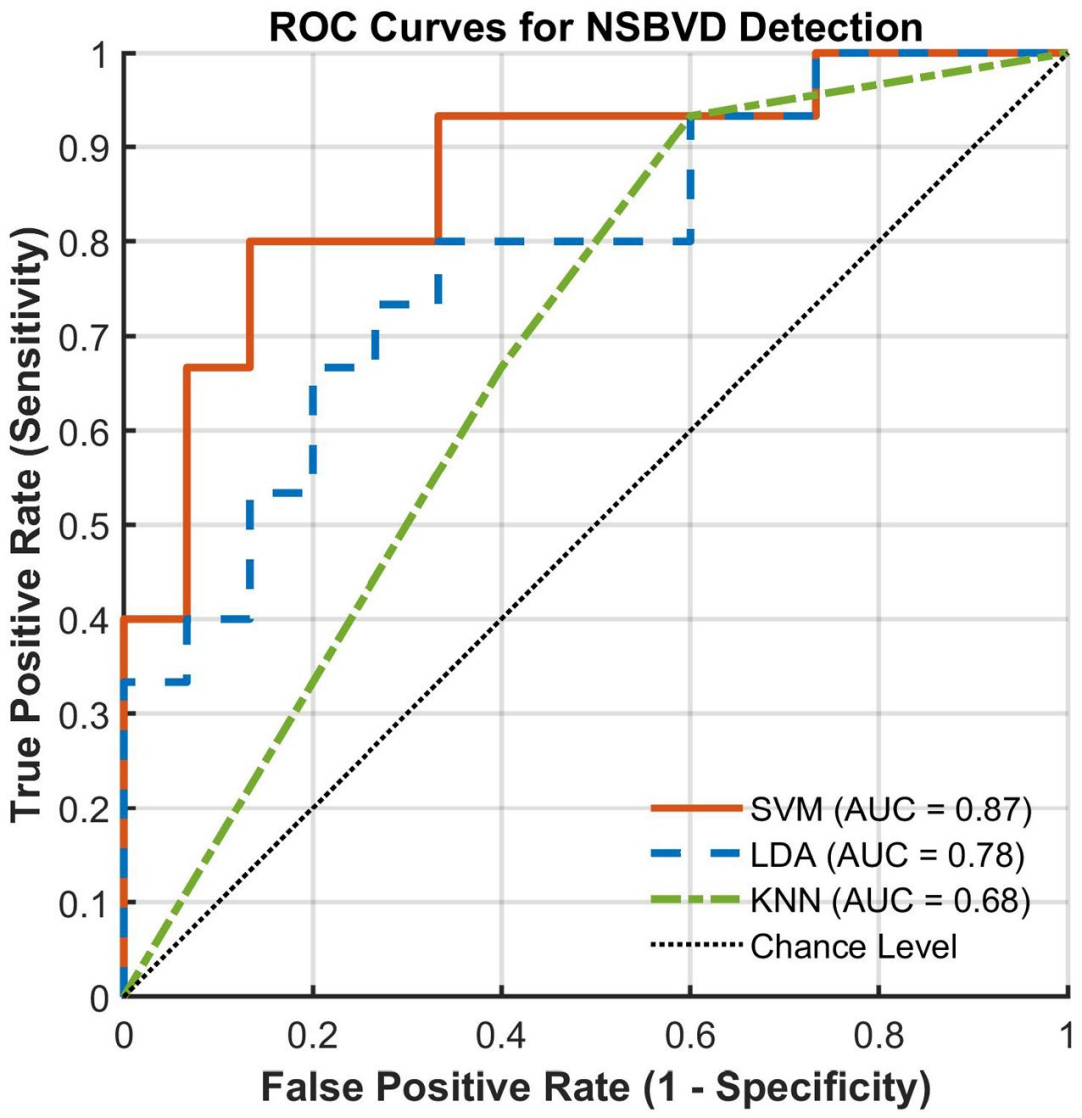
Receiver operating characteristic (ROC) curves comparing the diagnostic performance of SVM, LDA, and KNN classifiers.

The discriminative power of the models was further quantified using receiver operating characteristic (ROC) analysis (Figure 8). Consistent with the accuracy metrics, the SVM classifier achieved the highest AUC (0.8711), significantly exceeding the chance level. The steeper initial slope of the SVM ROC curve indicates superior sensitivity at low false positive rates, reinforcing its potential suitability for screening applications where missing a diagnosis is undesirable. Collectively, the superior performance of linear models (SVM and LDA) compared to the non-linear KNN approach suggests that the physiological features differentiating NSBVD patients from controls are likely separable by a linear decision boundary in the high-dimensional feature space.

## 4 Discussion

In the current study, we analyzed EEG signals collected from patients with NSBVD and healthy subjects in response to variable vectograms. In contrast to prior methodologies that relied primarily on subjective reports, we aimed to investigate the neurophysiological basis of NSBVD by examining brain topographic maps, time-frequency features, and the feasibility of machine learning for objective classification (Table 4).

During the initiation of convergence, we observed a distinct reversal in cortical polarity between groups. Specifically, 1 s after onset, the healthy control group exhibited a functional anterior-to-posterior gradient characterized by negative potentials in the frontal region and strong positive activation in the parietal and occipital regions. This distribution aligns with the established vergence network described by Alvarez et al. (2010, 2014), indicating efficient engagement of the posterior sensorimotor cortex to process disparity cues. Conversely, the NSBVD group displayed widespread positive potentials in the frontal region and negative potentials in the posterior regions. This transition from posterior to anterior processing implies that patients fail to utilize the dorsal visual stream, which is responsible for spatial action guidance (Milner and Goodale, 2006). Instead, they appear to rely on compensatory top-down executive control mediated by the prefrontal cortex (Friedman and Robbins, 2022). According to the neural efficiency hypothesis (Neubauer and Fink, 2009), this excessive frontal recruitment reflects a less efficient neural strategy in which the brain must expend higher cognitive effort to initiate movements that are automatic in healthy individuals.

As the task approached the fusion limit 1 s before the convergence break, the topographical differences continued to evolve. The NSBVD group exhibited sustained, deepening negative potentials in the parietal and occipital areas. This persistent negativity likely represents a breakdown in sensory integration or maladaptive cortical inhibition immediately preceding diplopia. According to the inhibition timing hypothesis (Klimesch, 2011), increased inhibitory signals often associated with alpha synchronization in visual areas may gate sensory processing. Unlike healthy controls who maintained a balanced state, the posterior visual networks of the patients appeared to disengage or be suppressed under high vergence load, potentially contributing to the loss of fusion (Widmer et al., 2018). Distinct neural strategies were also evident during the divergence block, where the interaction pattern shifted from compensatory effort to neural hypoactivation. Upon the onset of divergence, 1 s after onset, healthy controls demonstrated intense activation with deep negative frontal and strong positive posterior potentials reflecting the active recruitment of the divergence system. However, the NSBVD group presented a flattened topography with significantly attenuated amplitudes across the entire scalp. This lack of robust activation suggests that, unlike convergence, where they struggle with compensatory effort, patients with NSBVD exhibit a fundamental failure to mobilize the necessary cortical resources for divergence. This aligns with functional magnetic resonance imaging findings in binocular dysfunction, showing reduced peak velocities and cortical activity indicating a fragile vergence system unable to initiate varying disparity responses (Alvarez et al., 2010). Finally, approaching the divergence break 1 s before the divergence limit, the NSBVD group continued to show weak and nonspecific neural activity, whereas healthy controls maintained their activation patterns to maintain alignment. This pervasive hypoactivation implies that the divergence system in NSBVD patients is characterized by neural inertia or unresponsiveness. Previous neuroimaging studies have established that the magnitude of cortical activation in the frontal and parietal fields is significantly correlated with the peak velocity and precision of vergence movements (Alvarez et al., 2014). Therefore, the observed failure to mobilize cortical resources likely underpins the behavioral inability of the patient to sustain binocular alignment against uncrossed disparity (Alvarez et al., 2010; Widmer et al., 2018).

Complementing these topographical insights, the time frequency analysis provided a deeper understanding of the specific oscillatory strategies employed by the two groups. In the healthy control group, the divergence task analysis revealed a critical role for beta-band oscillations in sustaining binocular alignment. Specifically, a statistically significant cluster of elevated beta power was identified in the frontal region. Previous research suggests that beta activity is instrumental in the active maintenance of the current sensorimotor set and top-down cognitive control (Engel and Fries, 2010). The presence of this cluster suggests that as disparity stress increases, the healthy brain effectively mobilizes frontal executive resources to monitor and preserve fusion. Notably, this adaptive beta modulation was absent in the NSBVD group. This statistical absence reinforces the hypothesis that NSBVD patients suffer from a deficit in dynamically mobilizing cortical resources, preventing them from stabilizing the vergence system under high-demand conditions. Regarding the critical phase preceding the break in fusion, the comparisons between groups highlighted distinct pathological mechanisms depending on the vergence direction. During the convergence task, significant differences were localized to the frontal cortex, where statistical analysis identified prominent clusters of elevated power in both the theta and alpha bands before the break. The surge in frontal theta power is particularly illustrative. Consistent with the conflict monitoring hypothesis described by Ridderinkhof et al. (2004), frontal theta oscillations typically scale with the magnitude of cognitive conflict or error detection. In this context, the significant theta cluster suggests that the patient’s brain detects the looming disparity error as a high-conflict signal, triggering an excessive but ultimately inefficient compensatory effort. Simultaneously, the concurrent rise in frontal alpha power may reflect an attempt to suppress task-irrelevant processing to focus attention, yet the failure to maintain fusion suggests this resource allocation is maladaptive.

Conversely, the dysfunction during the divergence task appeared to be rooted in posterior sensory processing. The statistical results isolated a significant cluster of elevated alpha power in the parietal and occipital regions preceding the divergence break. Interpreting this specific alpha modulation requires a nuanced perspective. According to the gating-by-inhibition framework proposed by Jensen and Mazaheri (2010), alpha activity functionally inhibits information flow in specific cortical areas. Therefore, this significant posterior alpha cluster suggests that the patient’s visual system may be actively gating or suppressing the processing of divergent disparity cues. Alternatively, in line with the cortical idling hypothesis (Pfurtscheller et al., 1996), this activity could signal that the dorsal visual stream has disengaged due to an inability to process the uncrossed disparity effectively, giving up before the behavioral break occurs. Collectively, these findings delineate a dual dysfunction in NSBVD characterized by inefficient executive overdrive in the frontal cortex during convergence and a fundamental failure of posterior sensory integration during divergence.

To translate these neurophysiological findings into clinical utility, we integrated the identified rhythmic features into machine learning classifiers. Among the evaluated models, the linear SVM exhibited the most robust performance, achieving a classification accuracy of 76.67% and an area under the curve of 0.87. Crucially, the SVM model demonstrated a superior sensitivity of 80.00%. In the context of clinical screening, sensitivity is a paramount metric as it minimizes the risk of false negatives or missed diagnoses. Therefore, the high sensitivity observed here suggests that the identified EEG signatures possess a strong capability to correctly detect potential NSBVD cases. From a methodological perspective, the performance disparity between linear and nonlinear models offers intrinsic insights into the data structure. The superior performance of linear classifiers, specifically SVM and linear discriminant analysis, compared to the nonlinear K-nearest neighbors approach, suggests that the decision boundary between NSBVD and healthy neurophysiology is likely distinct and linearly separable in the high-dimensional feature space. This implies that the neurophysiological signatures of NSBVD are not merely subtle variations of the normal state but rather stable and identifiable pathological patterns. These results support the feasibility of using EEG signatures, specifically the differential modulation of alpha and beta rhythms, as reliable potential biomarkers for objective screening.

While the findings provide a valuable neurophysiological framework, several limitations should be noted to properly contextualize the results. First, the relatively small sample size in this pilot study limits the statistical power and the extent to which the findings can be generalized to the broader NSBVD population. Second, clinical heterogeneity within the patient group, particularly regarding specific subtypes such as convergence insufficiency versus excess, might introduce variability in the EEG signatures that the current model may not fully capture. Third, the cross-sectional design prevents definitive causal inferences regarding whether these neural deficits are the primary cause of the dysfunction or a secondary consequence of chronic visual stress. Finally, regarding the machine learning pipeline, although nested cross-validation was used to mitigate overfitting, the potential for bias in feature selection cannot be entirely ruled out in small datasets.

It is essential that these constraints be addressed to advance this field. Future investigations should prioritize recruiting larger, more diverse cohorts to enhance the robustness and reliability of the classification models. Furthermore, employing standardized diagnostic protocols to stratify patient subgroups would allow for a more detailed analysis of neural markers specific to different clinical conditions. From a methodological perspective, applying deep learning architectures directly to raw data could potentially uncover complex nonlinear features that traditional manual feature engineering might miss. Ultimately, longitudinal studies are required to clarify causal relationships and determine if vision therapy can modify these neural signatures, thereby establishing them as objective indicators of treatment success.

## 5 Conclusion

This study investigated the neurophysiological basis of NSBVD using EEG and machine learning. We identified distinct neural patterns characterized by reduced posterior activity and increased frontal activity. An SVM classifier based on these features distinguished patients from controls with 76.67% accuracy. These findings suggest that neural patterns are potential biomarkers for binocular dysfunction, demonstrating the feasibility of objective screening tools to complement traditional assessments.

To advance this pilot study, future work should focus on three key areas. First, recruiting larger cohorts of at least 100 participants is essential to validate the robustness of the machine learning models and ensure sufficient statistical power. Second, subsequent studies should use standardized symptom and diagnostic batteries to better stratify patient subgroups and address clinical heterogeneity. Finally, deep learning models, such as convolutional neural networks (CNNs), could be applied to raw time-frequency maps to uncover complex nonlinear features that traditional methods might miss.

## Data Availability

The datasets presented in this article are not readily available because they contain sensitive clinical EEG information that could potentially compromise the privacy of the participants. Requests to access the datasets should be directed to Qixuan Zhang, qixuan_zhang@foxmail.com.
